# Supplementary material for: Image Quality and Quantitative PET Parameters of Low-Dose [18F]FDG PET in a Long Axial Field-of-View PET/CT Scanner
Source: Diagnostics (Basel). 2023 Oct 18;13(20):3240. doi: 10.3390/diagnostics13203240 (PMC10606613; doi:10.3390/diagnostics13203240)
Supplement: Supplementary file 1 [file diagnostics-13-03240-s001.zip › diagnostics-2665214-supplementary.pdf]

Supplementary

Supplementary Table S1: Patient and dose characteristics and clinical information.

Patient, tumor type	Number of pathological lesions on STD examination (3.0 MBq/kg, 5 min, HS mode)	Patient characteristics				Dose characteristics		
		Body height in cm	Body weight in kg	BMI in kg/m^2	Age in years	Total dose of [^{18}F]FDG in MBq	Total dose of [^{18}F]FDG in MBq/kg	Effective dose of scan in mSv
#1 melanoma	1	1.69	60.4	21.15	85	164	2.72	3.12
#2 lymphoma	5	1.65	68.6	25.20	36	226	3.29	4.29
#3 lung cancer	2	1.67	83	29.76	63	264	3.18	5.02
#4 lymphoma	4	1.78	71	22.41	80	215	3.03	4.09
#5 esophageal cancer	5	1.7	76	26.30	68	237	3.12	4.50
#6 anal cancer	2	1.67	79	28.33	61	228	2.89	4.33
#7 lymphoma	2	1.58	53	21.23	92	129	2.43	2.45
#8 lymphoma	5	1.64	43	15.99	67	160	3.72	3.04
#9 CUP (adenocarcinoma)	5	1.7	69	23.88	76	245	355	4.66
#10 lung cancer	3	1.88	97.4	27.56	68	306	3.14	5.81
#11 colon cancer	2	1.8	93.6	28.89	41	300	3.21	5.70
#12 rectal cancer	4	1.84	96.1	28.38	64	279	2.90	5.30
#13 squamous cell carcinoma	2	1.71	49.5	16.93	43	157	3.17	2.98
#14 lung cancer	2	1.68	59.1	20.94	71	186	3.15	3.53
#15 lung cancer	2	1.65	74.3	27.29	76	242	3.26	4.60
#16 lymphoma	2	1.83	87.8	26.22	74	262	2.98	4.98
#17 lung cancer	3	1.64	57.7	21.45	53	177	3.07	3.36
#18 lung cancer	3	1.78	121	38.19	70	367	3.03	6.97
#19 lymphoma	1	1.83	71.5	21.35	27	214	2.99	4.07
#20 lymphoma	5	1.66	79.2	28.74	32	233	2.94	4.43
#21 CUP (squamous cell carcinoma)	1	1.89	88.5	24.78	80	268	3.03	5.09
#22 melanoma	4	1.6	57	22.27	69	201	3.53	3.82

#23 lung cancer	2	1.8	75.8	23.40	71	244	3.22	4.64
#24 melanoma	2	1.74	93.5	30.88	79	256	2.74	4.86
#25 melanoma	5	1.74	79.79	26.35	49	235	2.95	4.47
#26 lung cancer	5	1.76	76.6	24.73	64	211	2.75	4.01

CUP: Cancer of unknown primary; BMI: body-mass index.

Supplementary Table S2: Details of the CT imaging protocol

Scan parameters	
slice thickness	1 mm
pitch factor	0.3
twin beam dual energy AuSn120	
tube current	120 kV
tube voltage	Ref. mAs (using CARE Dose 4D)
contrast media	
portal venous phase (90 seconds p.i.)	80-140 mL Ultravist370 (Schering AG, Berlin, Germany) *
nativ	any contraindications for contrast media

*Weight-adapted intravenous injection at 1.0-2.5 mL/s, followed by a 40 mL saline flush

Supplementary Table S3: Results of two-way ANOVA between simulated dose reduction and sensitivity modes for uptake parameters and SNR values.

Parameter	Variable	df	F-value	p-value
SUV_{mean}	Simulated dose reduction	4	0	p=0.75
	Sensitivity mode	1	0	p=0.76
	Simulated dose*Sensitivity mode	4	0	p>0.99
SUV_{peak}	Simulated dose reduction	4	0	p=0.92
	Sensitivity mode	1	0	p=0.81
	Simulated dose*Sensitivity mode	4	0	p>0.99
SUV_{max}	Simulated dose reduction	4	0.24	p=0.91
	Sensitivity mode	1	4.17	p=0.0027
	Simulated dose*Sensitivity mode	4	4.04	p=0.0454
TBR	Simulated dose reduction	4	0	p=0.99
	Sensitivity mode	1	0	p=0.88
	Simulated dose*Sensitivity mode	4	0	p>0.99

SNR	Simulated dose reduction	4	1062	p<0.0001
	Sensitivity mode	1	316.0	p<0.0001
	Simulated dose*Sensitivity mode	4	18.35	p<0.0001

Supplementary Table S4: Multiple comparisons test for SUV_{max} and SNR.

Dependent variable (Bonferroni´s multiple comparison test)	Compared reconstructions	95% Confidence Interval	Adjusted p-value
SUV_{max}	3 MBq/Kg :HS Mode vs. 3 MBq/Kg :UHS mode	-2.993 to 4.100	>0.9999
	3 MBq/Kg :HS Mode vs. 1 MBq/Kg :HS Mode	-3.862 to 3.231	>0.9999
	3 MBq/Kg :HS Mode vs. 1 MBq/Kg :UHS mode	-3.169 to 3.923	>0.9999
	3 MBq/Kg :HS Mode vs. 0.5 MBq/Kg :HS Mode	-4.558 to 2.535	>0.9999
	3 MBq/Kg :HS Mode vs. 0.5 MBq/Kg :UHS mode	-3.481 to 3.612	>0.9999
	3 MBq/Kg :HS Mode vs. 0.25 MBq/Kg :HS Mode	-5.439 to 1.654	>0.9999
	3 MBq/Kg :HS Mode vs. 0.25 MBq/Kg :UHS mode	-4.196 to 2.896	>0.9999
	3 MBq/Kg :HS Mode vs. 0.125 MBq/Kg :HS Mode	-7.550 to -0.4575	**0.0161**
	3 MBq/Kg :HS Mode vs. 0.125 MBq/Kg :UHS mode	-5.416 to 1.677	>0.9999
SNR	3 MBq/Kg :HS mode vs. 3 MBq/Kg :UHS Mode	-3.790 to -2.375	**<0.0001**
	3 MBq/Kg :HS mode vs. 1 MBq/Kg :HS mode	3.355 to 4.770	**<0.0001**
	3 MBq/Kg :HS mode vs. 1 MBq/Kg :UHS Mode	1.260 to 2.674	**<0.0001**

SNR	3 MBq/Kg :HS mode vs. 0.5 MBq/Kg :HS mode	5.025 to 6.440	**<0.0001**
	3 MBq/Kg :HS mode vs. 0.5 MBq/Kg :UHS Mode	3.510 to 4.925	**<0.0001**
	3 MBq/Kg :HS mode vs. 0.25 MBq/Kg :HS mode	6.170 to 7.584	**<0.0001**
	3 MBq/Kg :HS mode vs. 0.25 MBq/Kg :UHS Mode	5.115 to 6.530	**<0.0001**
	3 MBq/Kg :HS mode vs. 0.125 MBq/Kg :HS mode	6.945 to 8.360	**<0.0001**
	3 MBq/Kg :HS mode vs. 0.125 MBq/Kg :UHS Mode	6.171 to 7.585	**<0.0001**
	3 MBq/Kg :UHS Mode vs. 1 MBq/Kg :HS mode	6.438 to 7.852	**<0.0001**
	3 MBq/Kg :UHS Mode vs. 1 MBq/Kg :UHS Mode	4.342 to 5.757	**<0.0001**
	3 MBq/Kg :UHS Mode vs. 0.5 MBq/Kg :HS mode	8.107 to 9.522	**<0.0001**
	3 MBq/Kg :UHS Mode vs. 0.5 MBq/Kg :UHS Mode	6.592 to 8.007	**<0.0001**
	3 MBq/Kg :UHS Mode vs. 0.25 MBq/Kg :HS mode	9.252 to 10.67	**<0.0001**
	3 MBq/Kg :UHS Mode vs. 0.25 MBq/Kg :UHS Mode	8,197 to 9.612	**<0.0001**
	3 MBq/Kg :UHS Mode vs. 0.125 MBq/Kg :HS mode	10.03 to 11.44	**<0.0001**
	3 MBq/Kg :UHS Mode vs. 0.125 MBq/Kg :UHS Mode	9.253 to 10.67	**<0.0001**
	1 MBq/Kg :HS mode vs. 1 MBq/Kg :UHS Mode	-2.803 to -1.388	**<0.0001**
	1 MBq/Kg :HS mode vs. 0.5 MBq/Kg :HS mode	0.9623 to 2.377	**<0.0001**
	1 MBq/Kg :HS mode vs.		

	0.5 MBq/Kg :UHS Mode	-0.5527 to 0.8620	>0.9999
	1 MBq/Kg :HS mode vs. 0.25 MBq/Kg :HS mode	2.107 to 3.522	**<0.0001**
	1 MBq/Kg :HS mode vs. 0.25 MBq/Kg :UHS Mode	1.052 to 2.467	**<0.0001**
	1 MBq/Kg :HS mode vs. 0.125 MBq/Kg :HS mode	2.883 to 4.297	**<0.0001**
	1 MBq/Kg :HS mode vs. 0.125 MBq/Kg :UHS Mode	2.108 to 3.523	**<0.0001**
	1 MBq/Kg :UHS Mode vs. 0.5 MBq/Kg :HS mode	3.058 to 4.473	**<0.0001**
	1 MBq/Kg :UHS Mode vs. 0.5 MBq/Kg :UHS Mode	1.543 to 2.958	**<0.0001**
SNR	1 MBq/Kg :UHS Mode vs. 0.25 MBq/Kg :HS mode	4.203 to 5.617	**<0.0001**
	1 MBq/Kg :UHS Mode vs. 0.25 MBq/Kg :UHS Mode	3.148 to 4.563	**<0.0001**
	1 MBq/Kg :UHS Mode vs. 0.125 MBq/Kg :HS mode	4.978 to 6.393	**<0.0001**
	1 MBq/Kg :UHS Mode vs. 0.125 MBq/Kg :UHS Mode	4.204 to 5.619	**<0.0001**
	0.5 MBq/Kg :HS mode vs. 0.5 MBq/Kg :UHS Mode	-2.222 to -0.8077	**<0.0001**
	0.5 MBq/Kg :HS mode vs. 0.25 MBq/Kg :HS mode	0.4373 to 1.852	**<0.0001**
	0.5 MBq/Kg :HS mode vs. 0.25 MBq/Kg :UHS Mode	-0.6173 to 0.7973	>0.9999
	0.5 MBq/Kg :HS mode vs. 0.125 MBq/Kg :HS mode	1.213 to 2.628	**<0.0001**
	0.5 MBq/Kg :HS mode vs. 0.125 MBq/Kg :UHS Mode	0.4384 to 1.853	**<0.0001**
	0.5 MBq/Kg :UHS Mode vs. 0.25 MBq/Kg :HS mode	1.952 to 3.367	**<0.0001**

	0.5 MBq/Kg :UHS Mode vs. 0.25 MBq/Kg :UHS Mode	0.8977 to 2.312	**<0.0001**
	0.5 MBq/Kg :UHS Mode vs. 0.125 MBq/Kg :HS mode	2.728 to 4.143	**<0.0001**
	0.5 MBq/Kg :UHS Mode vs. 0.125 MBq/Kg :UHS Mode	1.953 to 3.368	**<0.0001**
	0.25 MBq/Kg :HS mode vs. 0.25 MBq/Kg :UHS Mode	-1.762 to -0.3473	**<0.0001**
SNR	0.25 MBq/Kg :HS mode vs. 0.125 MBq/Kg :HS mode	0.06842 to 1.483	**0.0162**
	0.25 MBq/Kg :HS mode vs. 0.125 MBq/Kg :UHS Mode	-0.7062 to 0.7085	>0.9999
	0.25 MBq/Kg :UHS Mode vs. 0.125 MBq/Kg :HS mode	1.123 to 2.538	**<0.0001**
	0.25 MBq/Kg :UHS Mode vs. 0.125 MBq/Kg :UHS Mode	0.3484 to 1.763	**<0.0001**
	0.125 MBq/Kg :HS mode vs. 0.125 MBq/Kg :UHS Mode	-1.482 to -0.06727	**0.0165**
